# Worn surface topography and mathematical modeling of Ti-6Al-3Mo-2Sn-2Zr-2Nb-1.5Cr alloy

**DOI:** 10.1038/s41598-023-35883-1

**Published:** 2023-06-01

**Authors:** Ramadan N. Elshaer, Khaled M. Ibrahim, Ahmed Ismail Zaky Farahat

**Affiliations:** 1 Tabbin Institute for Metallurgical Studies, Cairo, Egypt; 2 Central Metallurgical R&D Institute, Cairo, Egypt

**Keywords:** Engineering, Materials science, Mathematics and computing

## Abstract

This study aims at investigating worn surface topography and mathematical modeling of annealed Ti-6Al-3Mo-2Sn-2Zr-2Nb-1.5Cr alloy using response surface methodology (RSM). The alloy was subjected to three different regimes in order to study their effect on mechanical properties. First regime was applying cold deformation by compression until 15% drop in height at room temperature. The second regime was performing solution treated on the deformed samples at 920 °C for 15 min then air-cooled (AC) to ambient temperature. Third regime was applying aging on the deformed and solution treated specimen for 4 hr at 590 °C followed by air-cooling. Three different velocities (1, 1.5, and 2 m/s) were adopted to conduct dry sliding wear according to the experimental design technique (EDT). Gwyddion and Matlab softwares were used to detect worn surface photographs analytically and graphically. Maximum hardness of 425 HV_20_ was obtained for AC+Aging specimen, while minimum hardness of 353 HV_20_ was reported for the annealed specimen. Applying aging process after solution treatment enhanced considerably the wear property and this enhancement reached 98% as compared to the annealed condition. The relationship between input factors (hardness & velocity) and responses (Abbott Firestone zones) was demonstrated using analysis of variance (ANOVA). The best models for Abbott Firestone zones (high peaks, exploitation, and voids) produced accurate data that could be estimated for saving time and cost. The results showed that the average surface roughness increases with increasing sliding velocity for all conditions except AC+Aging condition where the average surface roughness decreased with increasing sliding velocity. The results revealed that at low velocity and hardness, the material gives the highest exploitation zone (86%). While at high velocity and hardness, the material gives the lowest exploitation zone (70%). In general, the predicted results of mathematical model showed close agreement with experimental results, creating that models could be utilized to predict Abbott Firestone zones satisfactorily.

## Introduction

TC21 Ti-alloy has high strength, hardness and toughness which is considered a revolutionary type of α+β titanium alloys. Aerospace industry has successfully utilized TC21 alloy, which has the following chemical formula: Ti-6Al-3Mo-2Sn-2Zr-2Nb-1.5Cr-0.1Si, to build essential components such as landing gear connection boxes and airfoil joints^1–3^. By controlling microstructure and workability, depending on thermomechanical and heat treatments, two phases (α/β) titanium alloys can achieve a better balance of mechanical and physical characteristics. Researchers were also interested in titanium alloys with equiaxed microstructures because of their high strength and superior fatigue characteristics. However, its usage is constrained by its low hardness and weak tribological behavior^4–7^. Post-deformation heat treatment processes can be applied for enhancing the tribological behavior of titanium alloys^1,8^.

Material ratio curve (Abbott Firestone curve) is a term for one of the metrics used to define surface roughness and profile. This curve exhibits the relation between protrusions (areas with the material) and depressions (areas devoid of material). One benefit of using Abbott-Firestone curve is to examine surfaces that can imitate the effects of wear and the running-in process. Additionally, this curve provides details about the void volumes and materials that characterize the surface topography. Recently, it may be helpful for defining and employing functional criteria in 3D research^9,10^. A useful characteristic for evaluating the functional qualities of surfaces and their applications is the Abbott-Firestone curve.

Some authors have asserted in earlier publications^11^ that the Abbott Firestone curve would characterize the initial and worn surfaces more accurately than surface roughness (R_a_), a claim that is supported by Torrance^12^. The deep voids may be altered or not, affecting, for example, the ability of the surfaces in contact to lubricate. A tribological technique may eliminate the peaks, resulting in a different texture being placed on the resulting plateau. When multiple types of wearing are occurring simultaneously, the Abbott Firestone curve can be used for gauging the impact of synergistic processes, such as tribological ones. Examining this curve while the triboelements exploitation may provide insight into the likelihood that the surface may change in the near future. In order to investigate the textural quality of gear teeth, Sosa et al.^13^ conducted 2D studies of the Abbott Firestone curve. In a different work, Sosa et al.^14^ examined the running-in process of the tooth and found that the voids appear to remain unaltered while the asperity peaks are worn off. Then they highlighted the variations in the peak zone (up to 30%) of the 2D Abbott-Firestone curves. By contrasting the Abbott-Firestone curves for the afflicted and unaffected zones of the femoral head composed of advanced ceramics, Affatato et al.^15^ were able to identify the worn surface. When examining how different surface topographies affect tribological properties, Mathia and Pawlus provided examples and emphasized the importance of surface characterization and testing^16^. According to Bruzzone et al.^17^, researching the connections between surface topography, function, and application is a particularly challenging undertaking that places a special emphasis on tribology. Kara et al.^18^ investigated the effects of shallow and deep cryogenic treatment on Sleipner cold work tool steel in terms of microhardness, microstructure, coefficient of friction, and wear rate. Elshaer et al.^19^ investigated the surface texture of carbon steel machine elements using Abbott Firestone curve.

Nowadays, design of experiments (DOE) techniques such as response surface methodology (RSM), Taguchi, and factorial design (FD) methods are frequently used in place of the time-consuming and expensive one-factor-at-a-time experimental technique. RSM uses modeling techniques to establish the relationship between experiment input and output variables. This method has gained popularity in engineering problems and has been heavily used in the characterization of issues where input elements have an impact on the certain performance of output components. When compared to other optimization techniques, RSM provides quantitative measures of potential factor interactions. RSM is the best strategy to apply when dealing with multi-variable replies. This method dramatically reduces the number of trials required to respond to a model. The use of RSM to enhance process characteristics was investigated by the authors^20–23^. In particular, when evaluating material qualities, you’ll need a mathematical model that can forecast the response output based on the effects of various process variables. Mechanical and tribological properties can be predicted using DOE, regression analysis and analysis of variance (ANOVA)^20,21,24,25^.

Chauhan and Dass^26^ used RSM to investigate how load, speed, and sliding distance affected the wear resistance of titanium alloy (Grade 5). They noticed that the wear rate increases with an increase in the typical applied load and speed and drops with an increase in the sliding distance and a decrease in speed. They came to the conclusion that the measured and predicted values are adequately close to one another, indicating that the proposed quadratic model can be employed effectively for forecasting the particular wear rate of titanium alloy with a 95% confidence level. Using RSM, Elshaer et al.^20^ assessed how pressure and velocity affected low-carbon steel’s Abbott Firestone zones and wear behavior. The impact of load (P) and linear sliding speed (V) on the wear behavior and friction coefficient of 13Cr5Ni2Mo steel was investigated by Meddah et al.^27^. There is a shortage in study of the surface roughness of Ti alloys. Therefore, this study aims at investigating the worn surface topography after wear testing of TC21 Ti-alloy using Gwyddion and Matlab softwares. In addition create a model for predicting Abbott Firestone zones (high peaks, exploitation, and voids) as a function of hardness and sliding velocities during the wear test using RSM.

## Experimental work

### Materials and specimen preparation

**Figure 1:**
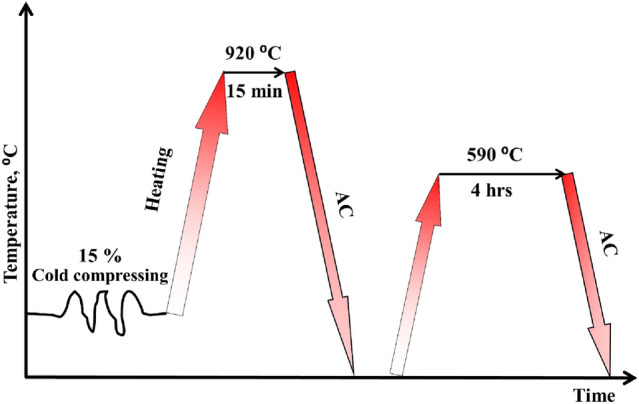
Cycles of three different regimes.

In the present work, the material used was an annealed TC21 Ti-alloy bar with 7 mm diameter and 140 mm long. The transformation temperature, β transus temperature, (T_β_) was previously determined at approximately 955 °C^28^. The alloy under investigation has the following chemical compositon; Ti-6.5Al-3Mo-1.9Nb-2.2Sn-2.2Zr-1.5Cr-0.09Si (wt.%). The alloy was subjected to three different regimes to study their effect on mechanical properties. First regime after annealing was cold compressing until 15% drop in height at ambient temperature using a universal testing machine and a stroke strain rate of 0.01 s^−1^. The specimens were prepared with dimensions of 7 mm in diameter and 11.5 mm length for cold compression test. While second regime was cold deformation followed by solution treated at 920 °C for 15 min then air-cooled (AC) to ambient temperature. Third regime after cold deformation and solution treatment was aging for 4 hr at 590 °C followed by air-cooling. There are 3 different rigimes used in this work Fig. 1.

Vickers hardness measurements were carried out in accordance with ASTM E92-16 Standard, using a load of 20 kg force for a dwell duration of 15 sec. Five readings were taken, and the mean value was reported. According to ASTM G99-17 Standard, the wear test was carried out utilizing pin-on-ring tribometer testing apparatus in a dry state at ambient temperature. Three time wear experiments were repeated and average was taken. The spinning hardened stainless steel ring (wear tool) had an outer diameter of 73 mm and a surface hardness of 63 HRC. The wear specimens had a cylindrical shape with 7.9 mm in diameter and 10 mm length. The ring surface was polished prior to each test using various emery sheets with a grit size of 1000. With a steady applied load of 50 N for 5 min and various linear sliding speeds of 1, 1.5, and 2 m/s were used. Prior to wear testing, the sample’s weight was determined using an electronic scale with 0.1 mg precision. FESEM was used to examine the worn surfaces of wear-tested specimens. Gwyddion and Matlab softwares have been used to process the worn surface photographs analytically and graphically. Surface roughness and Abbott Firestone curves were also produced using statistical analysis and Excel software.

### Statistical analysis using RSM

Design Expert-V13 was used for evaluating hardness and worn surface microscopy data. Software for designing experiments and performing statistical analyses uses the Response Surface Methodology (RSM). The term “RSM” refers to a group of statistical and mathematical approaches for modeling and analyzing issues where the objective is to maximize a response that is affected by a number of variables. Therefore, it is considered as a great method for evaluating industrial difficulties. There are three models for Abbott Firestone zones (high peaks, voids, and exploitation). The correlation between response and input variables in RSM can be formullated follows:

1 $$ {\text{Y }} = {\text{ f }}\left( {{\text{A}},{\text{ B}}} \right), $$

where f is the response function, A is the hardness, B is the velocity, and Y is the desired response.

The scientists used a polynomial design of experiments of type Pn, where “n” denotes the number of variables (hardness and velocities of wear test) and “P” denotes the number of levels (− 1, 0, +1). In light of this, 3^2^ = 9 trial tests must be carried out as a minimum for each condition. The Experimental Central Composite Design (CCD) was used in this investigation, and there were 13 runs with three levels and two variables (Table 1). A value of 0 denotes the average value, a value of +1 is the maximum limit, and a value of − 1 is the minimum limit of the parameters. The following formula can be used to construct the second-order polynomial regression equation, which was used to build a mathematical model and has two parameters.

2 $$ {\text{R }} = {\text{ b}}_{0} + {\text{ b}}_{{1}} {\text{A }} + {\text{ b}}_{{2}} {\text{B }} + {\text{ b}}_{{3}} {\text{AB }} + {\text{ b}}_{{4}} {\text{A}}^{{2}} + {\text{ b}}_{{5}} {\text{B}}^{{2}} + {\text{ b}}_{{6}} {\text{A}}^{{2}} {\text{B}}, $$

where b_0_ is the response average, b_1_, b_2_……b_7_ are response coefficients, A is hardness, B is velocity, and R is estimated.

**Table 1:** Experimental central composite design (CCD).

Std	Run	Factor 1	Factor 2
		A: hardness, HV	B: velocity, m/s
1	12	− 1	− 1
2	13	1	− 1
3	3	− 1	1
4	2	1	1
5	5	− 1	0
6	8	1	0
7	7	0	− 1
8	4	0	1
9	11	0	0
10	6	0	0
11	9	0	0
12	1	0	0
13	10	0	0
Variables	Levels		
	− 1	0	1
Hardness, HV	353	389	425
Velocity, m/s	1	1.5	2

## Results and discussion

### Hardness

**Figure 2:**
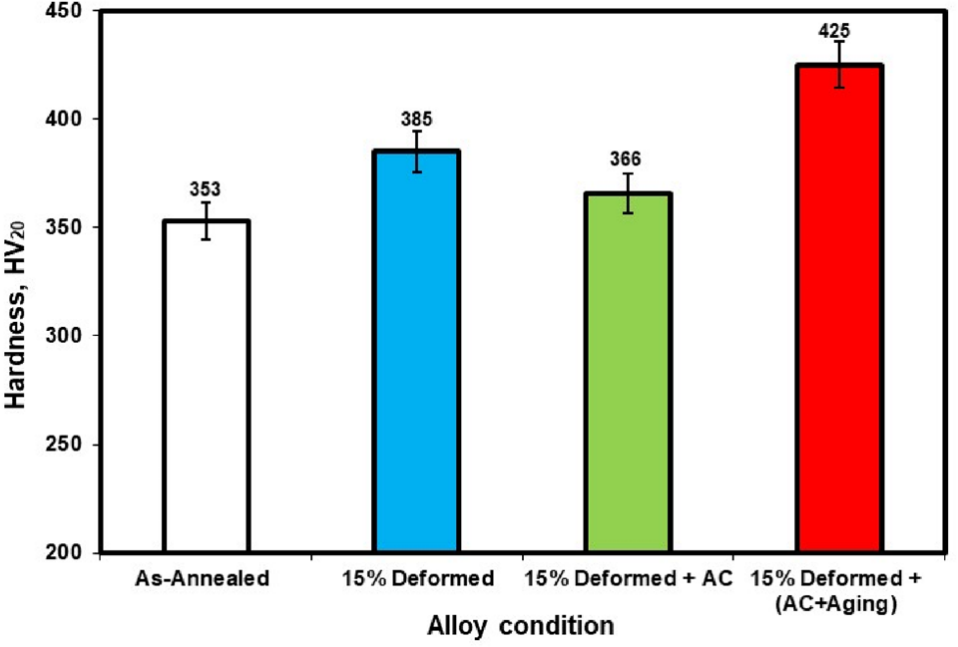
Hardness of annealed, cold-deformed, air-cooled, and both air-cooled and aged.

Figure 2 depicts the variance in hardness for various conditions (annealed, cold-deformed, air-cooled, and both air-cooled and aged). The hardness increased from 353 HV_20_ (annealed specimens) to 385 HV_20_ (deformed specimens). This indicates that strain hardening, as well as strengthening mechanisms, result in an increase in hardness of roughly 9% as a result of applying 15% cold deformation. In comparison to the cold deformed specimens, the hardness dropped after solution treating specimens to 366 HV_20_. However, for air-cooled and aged (AC+Aging) specimens, the hardness increased again to 425 HV_20_.

### Wear property

**Figure 3:**
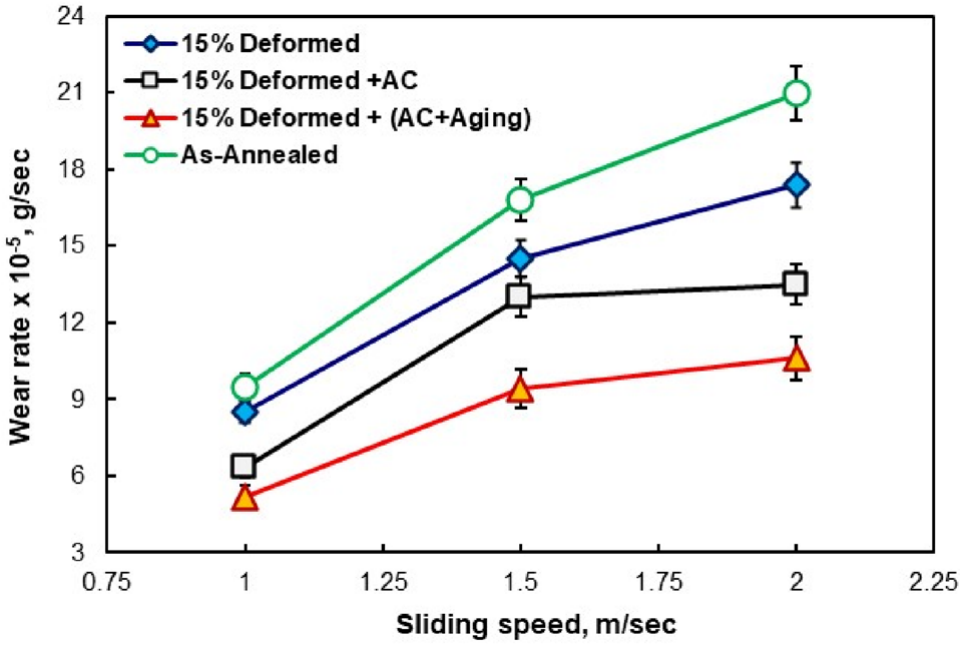
Wear rate of annealed, cold-deformed, air-cooled, and both air-cooled and aged.

Figure 3 shows the relationship between wear rate and sliding speed, which ranges from 1, 1.5, and 2 m/s with various TC21 Ti-alloy conditions (annealed, cold-deformed, AC, and AC+Aging). For all conditions, as the sliding speed rose, the rate of wear increased. The deformed specimens have the minimum wear rate compared to annealed ones. This is due to its high hardness value of 385 HV_20_ compared to annealed specimens (353 HV_20_). Applying 15% deformation on the annealed specimens plays an important role in increasing hardness of the deformed specimens and then improving the wear property. Therefore, applying 15% deformation to annealed specimens is crucial for raising hardness of the deformed specimens and thus enhancing their wear resistance. The lowest wear rate was recorded for cooled and aged specimens owing to their high hardness of 425 HV_20_. By applying the aging process, air-cooled specimens have an improvement of about 38% (at 1.5 m/s). Thus, it may be concluded that aging process following solution treatment (AC+Aging) can significantly improve the wear property of TC21 Ti-alloy. This means that an increase of up to 98% when comparing air-cooled to annealed specimens. The hardness and wear characteristics of the studied TC21 Ti-alloy exhibit a strong link with each other, in accordance with the Archard theory. These results were in agreement with the results of Ibrahim et al.^8^.

#### Worn surfaces

**Figure 4:**
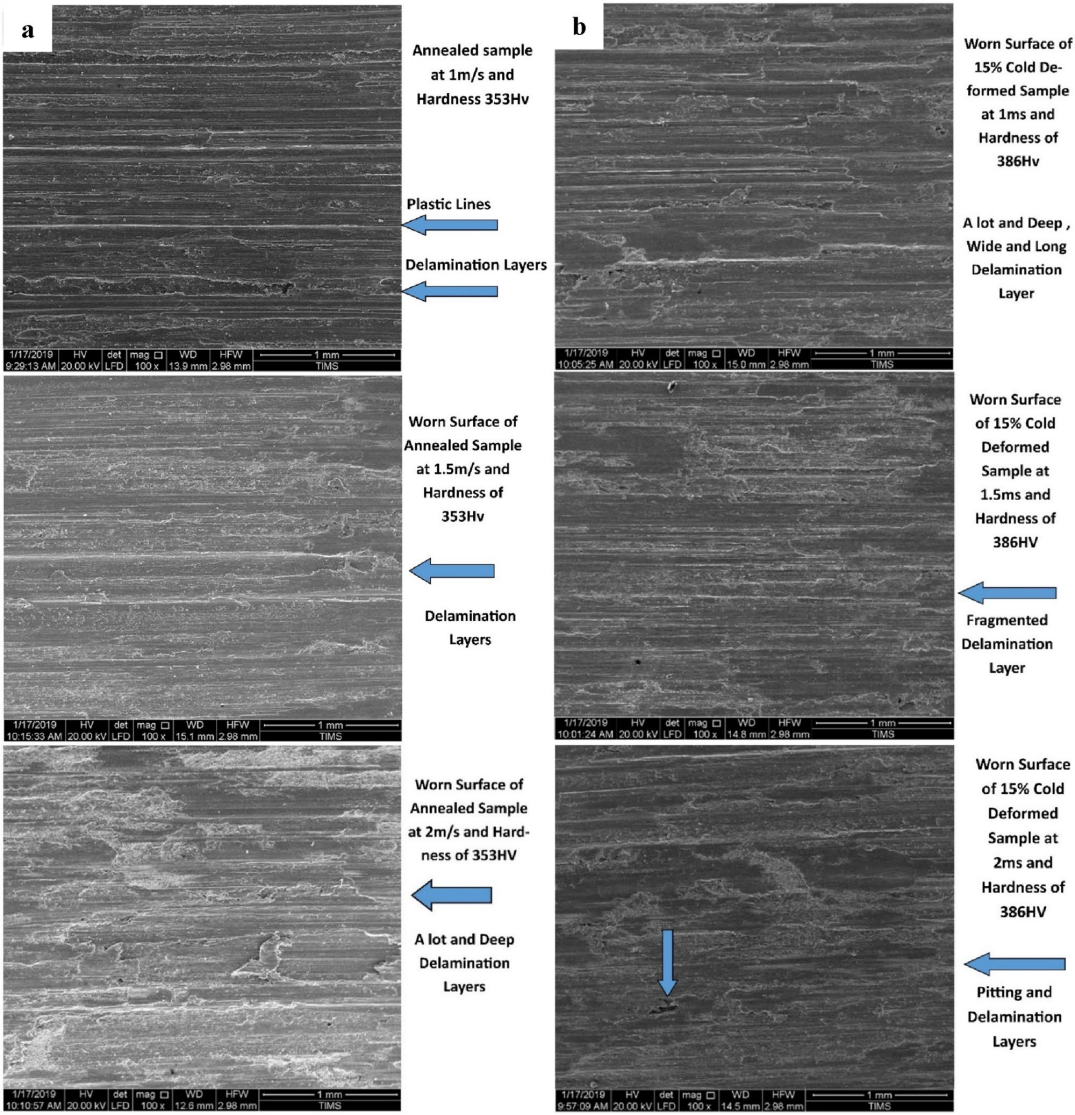
Worn surfaces of (**a**) annealed and (**b**) cold-deformed specimens.

**Figure 5:**
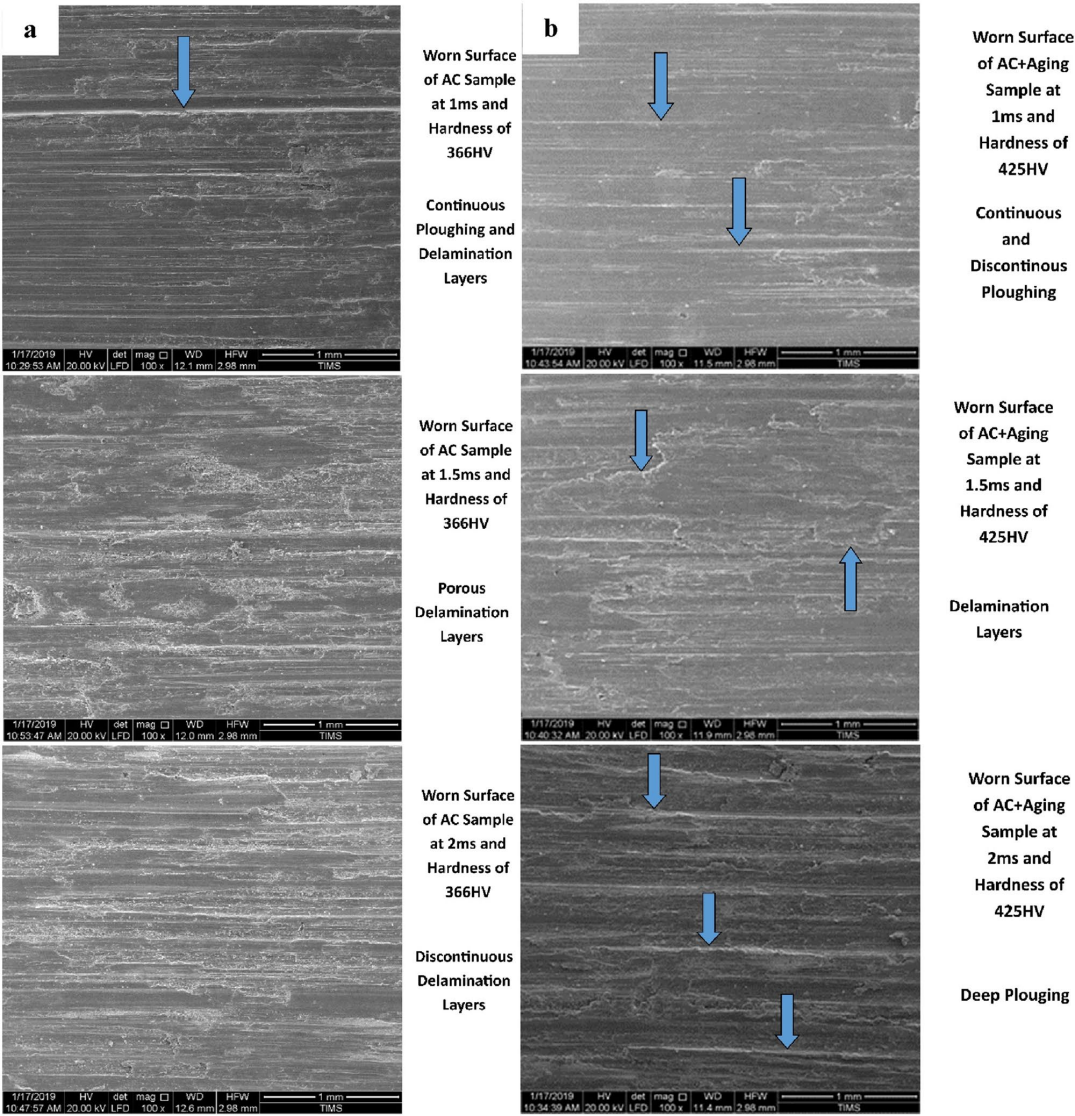
Worn surfaces of (**a**) air-cooled & (**b**) air-cooled and aged specimens.

Figures 4 and 5 show the worn surfaces of some selected wear specimens tested at an applied constant load of 50 N for 5 min and different velocities (1, 1.5, and 2 m/s) at various conditions (annealed, cold-deformed, air-cooled, and both air-cooled and aged). In most examined specimens, there are signs of plastic deformation on the worn surfaces. Particularly at a low sliding velocity of 1 m/s, continuous sliding markings with plastically distorted scratches or grooves can be also detected over the wear tracks. The worn surfaces at high sliding velocity (2 m/s) obtained highly plastic deformation or ploughing.

Figure 4a demonstrates annealed worn surface at the applied constant load of 50 N for 5 min. and different velocities (1, 1.5, and 2 m/s). At 1 m/s, the worn surface exhibits plastic lines, deep scratches, and lamination layers. While by increasing velocity to 1.5 m/s, the worn surface produces only more dense delamination layers. On the other hand at 2 m/s, the worn surface obtains deep gouging layers with deeper scratches. Figure 4b shows the cold deformed worn surface at the applied constant load of 50 N for 5 min at different velocities (1, 1.5, and 2 m/s). At 1m/s, the worn surface exhibits more, deep, long and wide delamination layers. By increasing velocity to 1.5 m/s, the worn surface produces fragmented delamination layers due to the tearing effect. On the other hand at 2 m/s, the worn surface shows pitting and delamination layers.

Figure 5a shows the air-cooled (AC) worn surface at the applied constant load of 50 N for 5 min at different velocities (1, 1.5, and 2 m/s). At 1 m/s, the worn surface exhibits continuous ploughing and delamination layers. In addition, it showed a smooth, flat worn surface with few small scratches. While by increasing velocity to 1.5 m/s, the worn surface produces porous delamination layers owing to the tearing effect. On the other hand at 2 m/s, the worn surface suffers discontinuous delamination layers along a groove on the worn surface. However, Fig. 5b shows the air-cooled and aged (AC+Aging) worn surface. At 1 m/s, the worn surface exhibits continuous and discontinuous ploughing. With increasing velocity to 1.5 m/s, the worn surface produces delamination wear mechanism due to the tearing effect. On the other hand at 2 m/s, the worn surface shows from deep ploughing.

#### Surface roughness of the worn specimens

**Figure 6:**
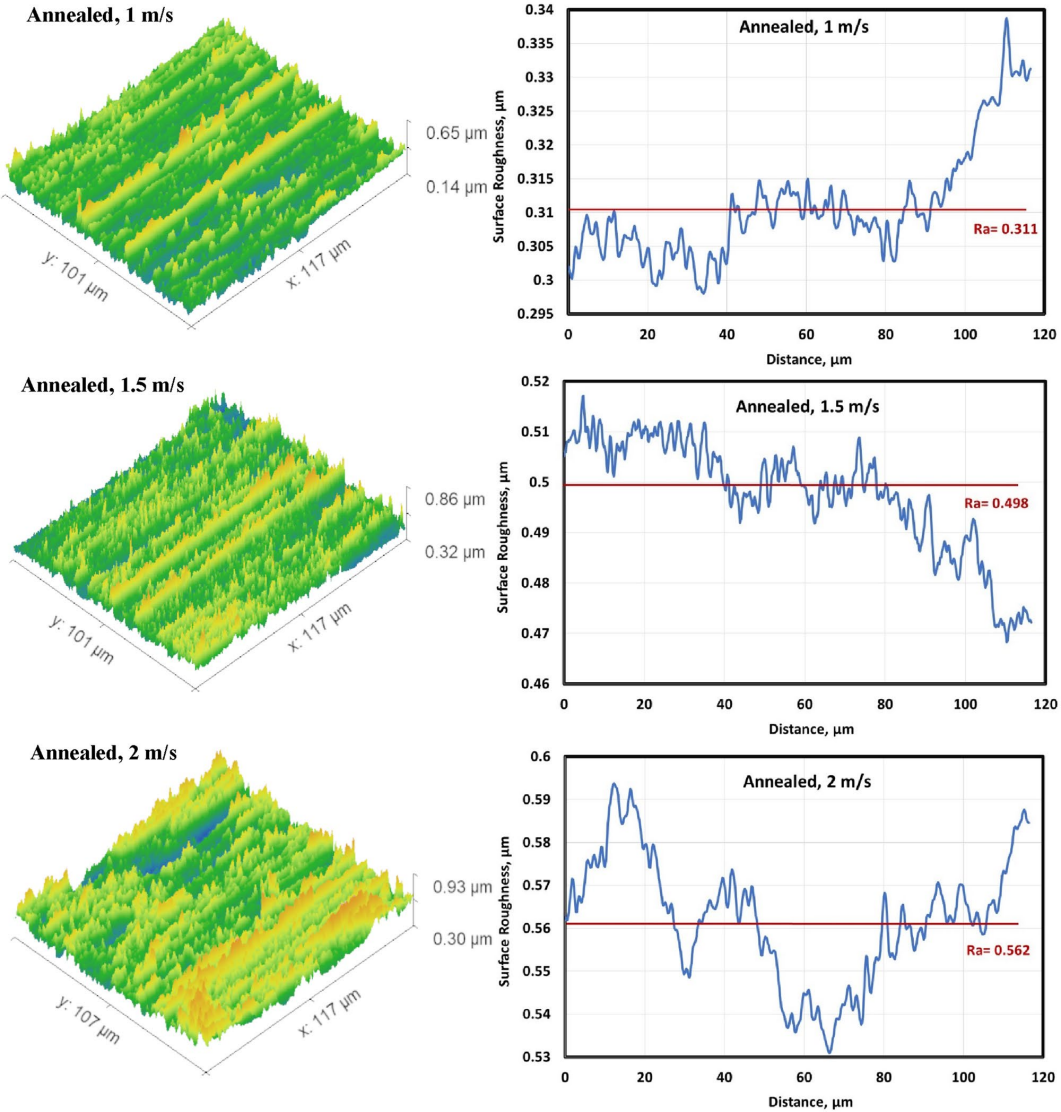
Surface roughness profile of annealed specimens.

**Figure 7:**
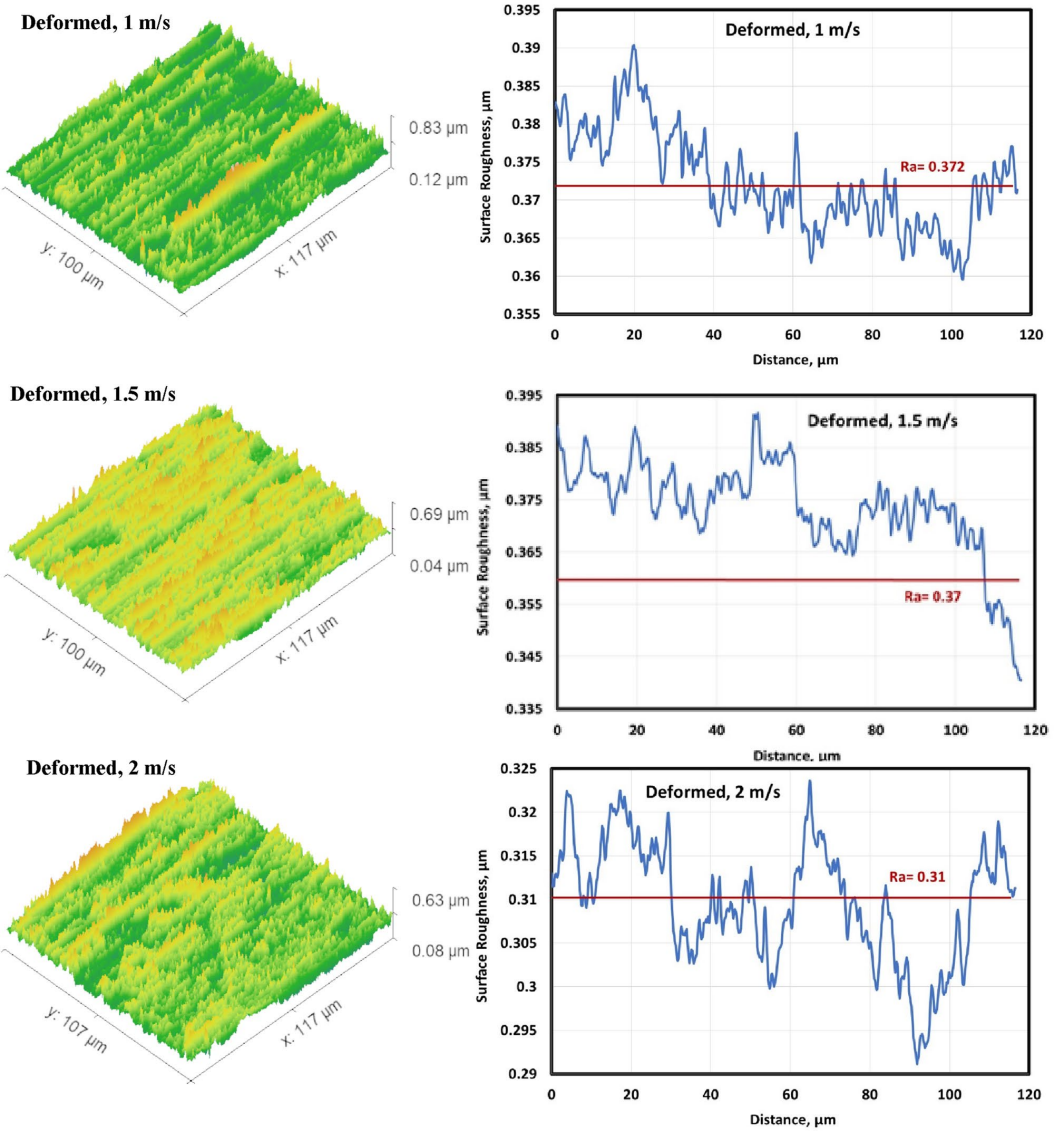
Surface roughness profile of cold deformed specimens.

**Figure 8:**
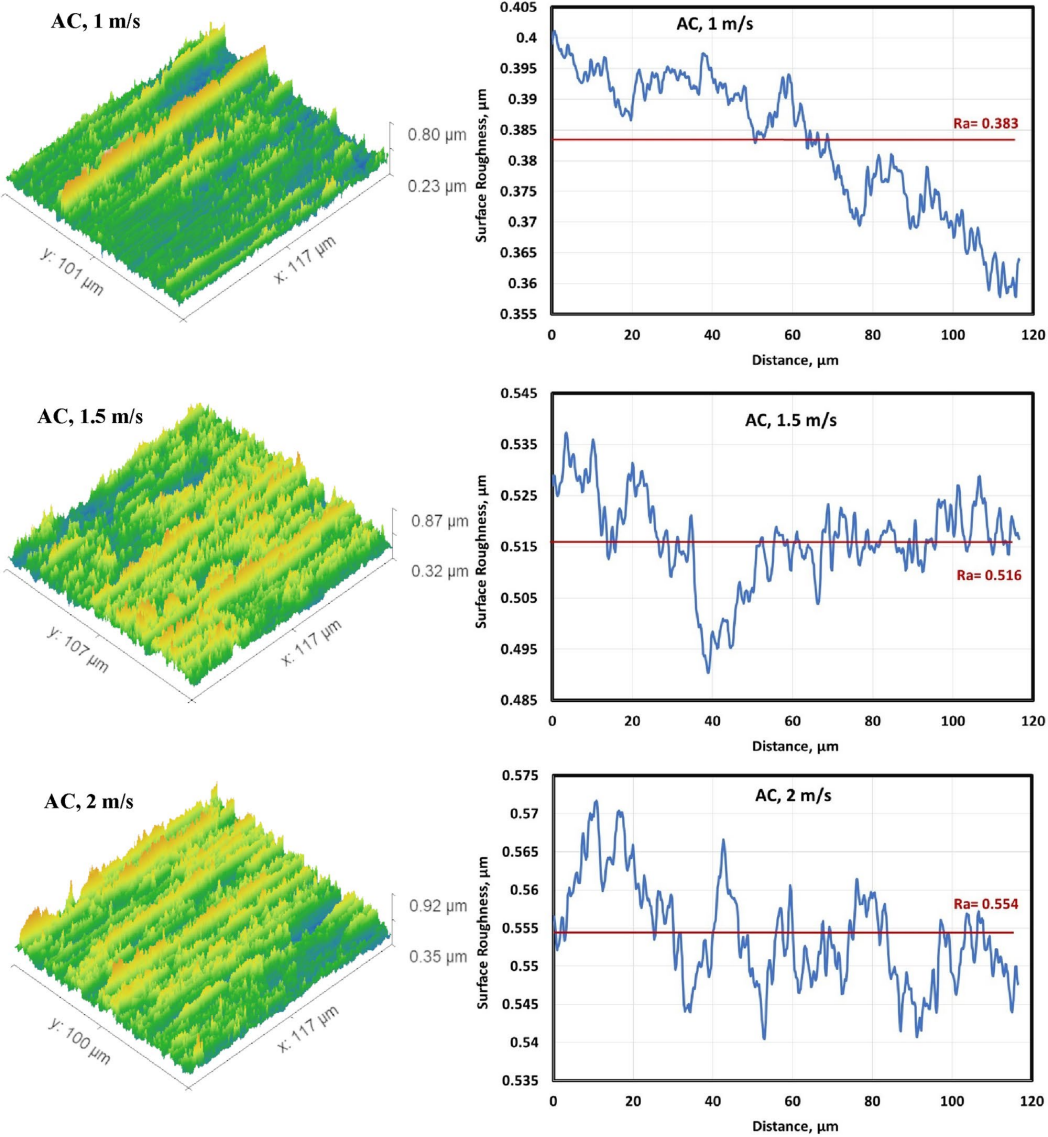
Surface roughness profile of air-cooled specimens.

**Figure 9:**
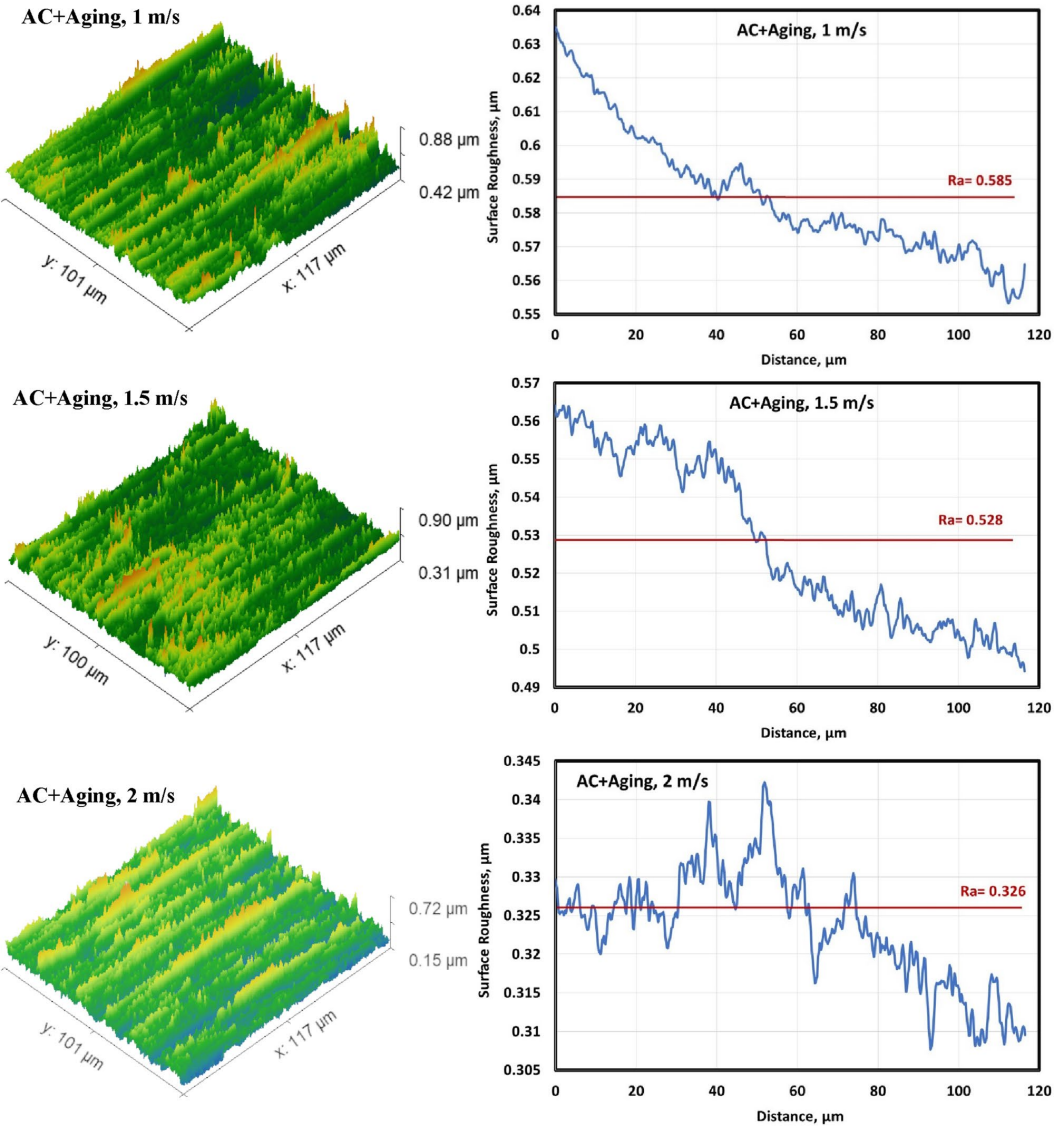
Surface roughness profile of air-cooled and aged specimens.

Figures 6, 7, 8 and 9 show surface roughness of the worn specimens for various metallurgical conditions (annealed, cold-deformed, air-cooled, and both air-cooled and aged). These figures demonstrate the different surface roughness profiles. Profile of the surface was evaluated on the worn surfaces, which are some of the parameters for the assessment of the surface quality after wear. It is clear that the average surface roughness profile is directly related to velocity and material conditions. The average surface roughness increases with increasing sliding velocity for all conditions except AC+Aging condition, where the average surface roughness decreases with increasing sliding velocity. However, these profiles can not quantitively judge the surface texture in detail. Therefore, it was important to adopt powerful and simple techniques such as Abbott Firestone technique to quantitively recognize the surface roughness profiles due to different velocities and material conditions.

**Figure 10:**
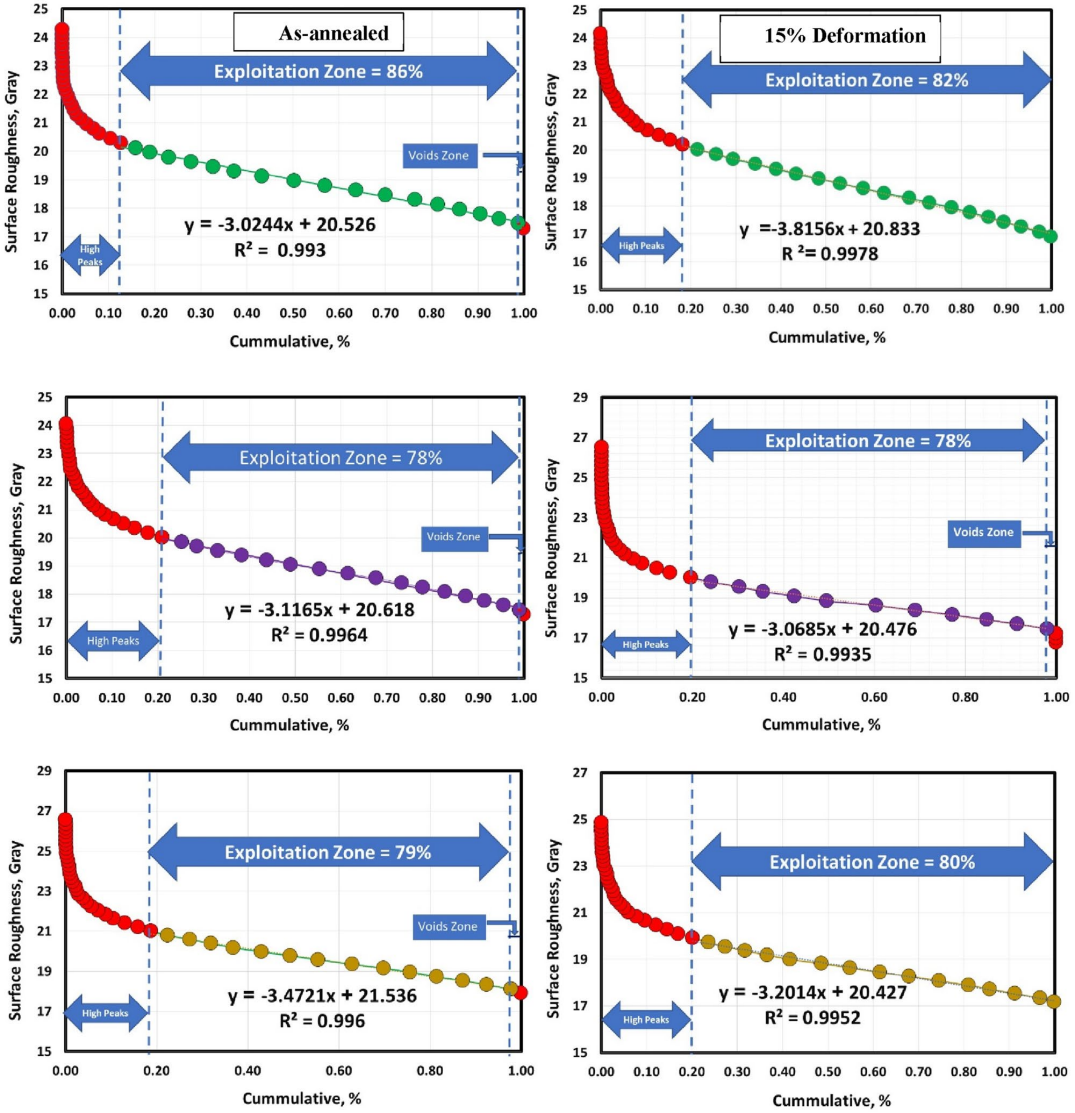
Abbott Firestone curves of annealed and cold deformed conditions.

**Figure 11:**
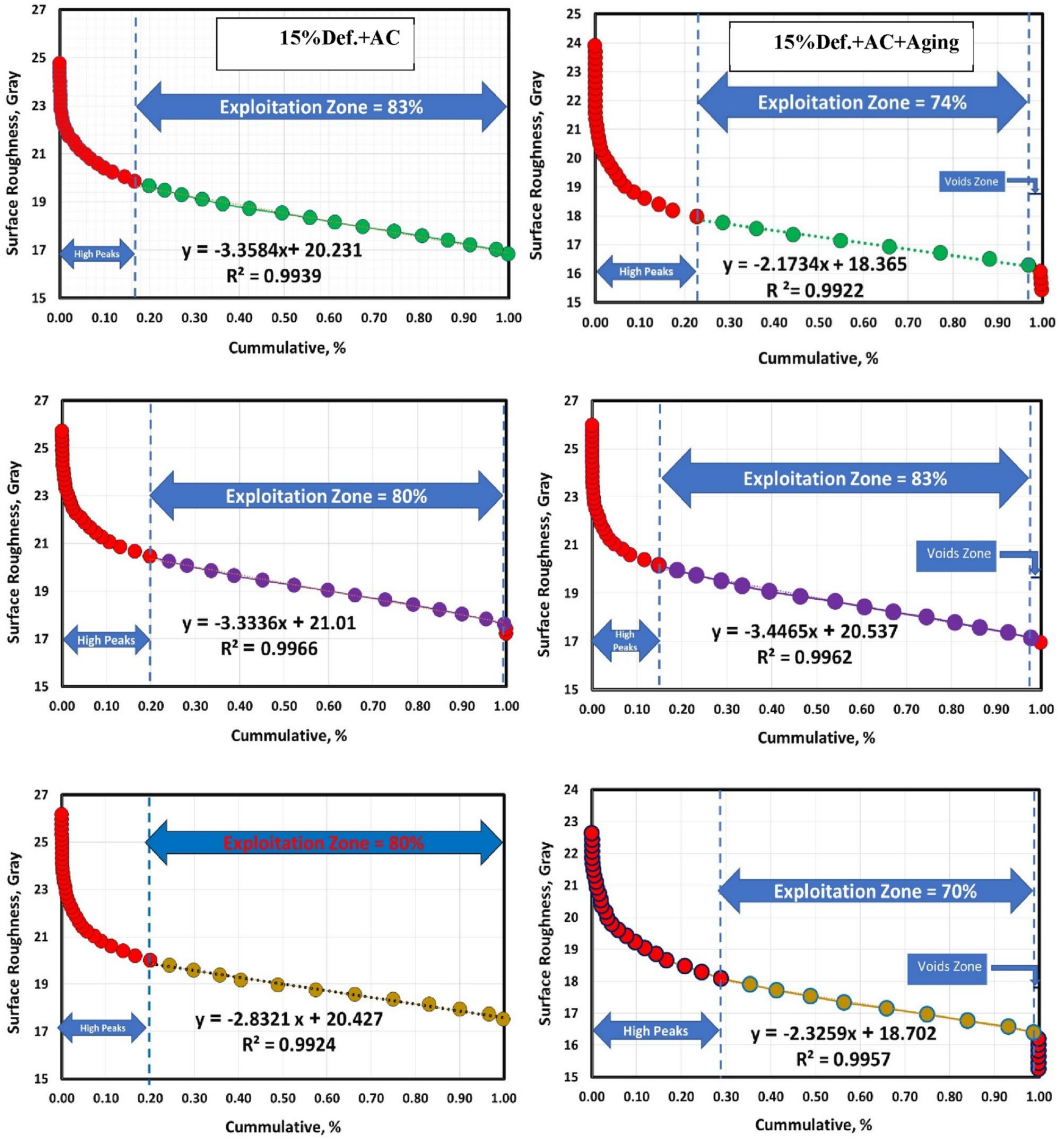
Abbott Firestone curves of AC and AC+Aging conditions.

Figures 10 and 11 show the Abbott Firestone curves for various sample conditions (annealed, cold-deformed, air-cooled, and both air-cooled and aged). Most curves can be divided into three zones. Zone I is called the high peak where this zone approximately increases with increasing sliding speed in most conditions. Zone II is called the exploitation zone where this zone approximately decreases with increasing sliding speed. Finally, zone III is called voids zone. Another curves could be divided into two zones, high peak and exploitation zone where the voids zone disappeared. Further details of the three zones (high peak, exploitation, and voids) values for the annealed, cold-deformed, air-cooled, and both air-cooled and aged are given in Table 2.

**Table 2:** High peak, exploitation, and voids values for various metallurgical conditions.

Conditions		Zones		
		High peaks %	Exploitation %	Voids %
As-annealed	1 m/s	13	86	1
	1.5 m/s	21	78	1
	2 m/s	19	79	2
15% cold def.	1 m/s	18	82	0
	1.5 m/s	20	78	2
	2 m/s	20	80	0
15% cold def. +AC	1 m/s	17	83	0
	1.5 m/s	19.6	80	0.4
	2 m/s	20	80	0
15% cold def. +(AC+Aging)	1 m/s	23	74	3
	1.5 m/s	15	83	2
	2 m/s	29	70	1

**Figure 12:**
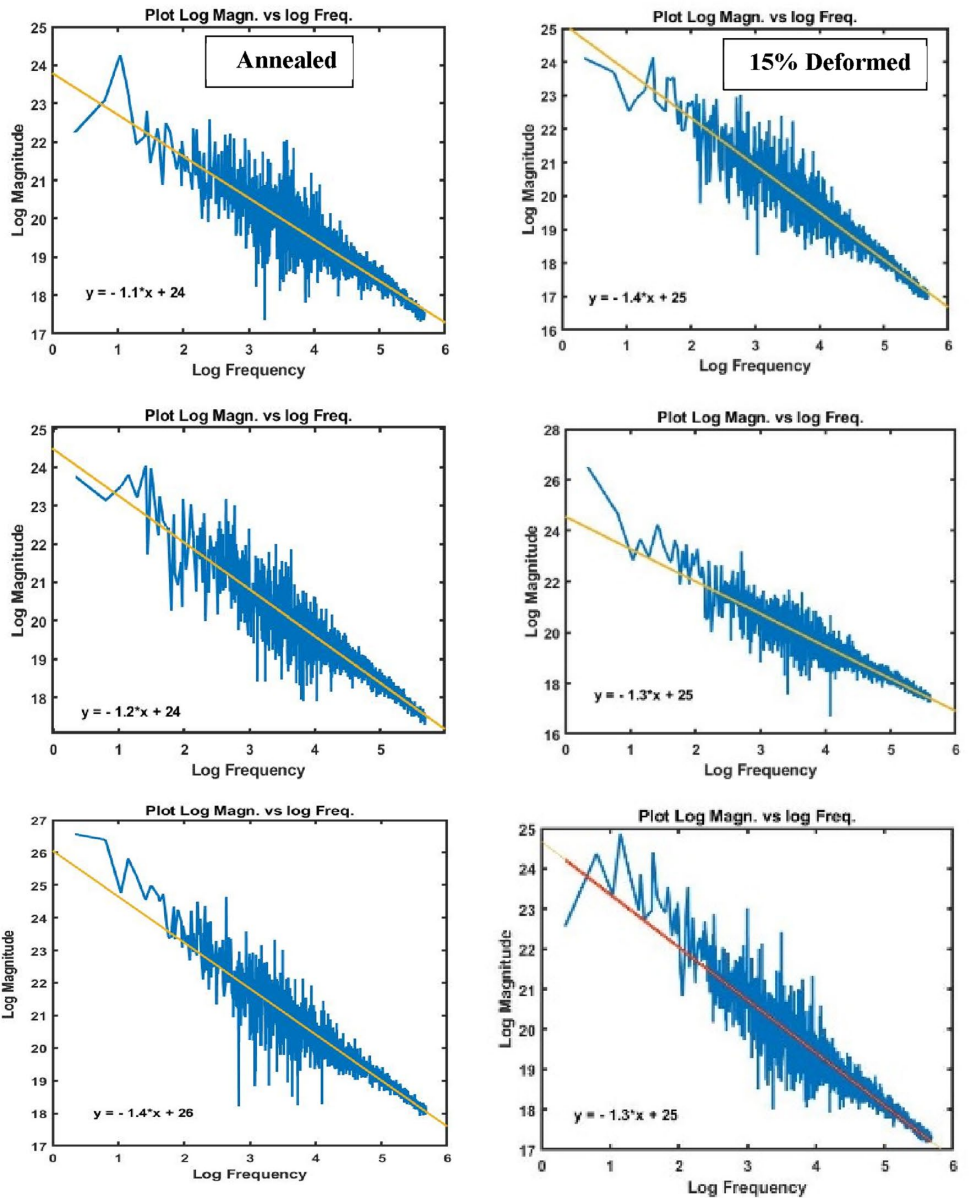
Different peaks of worn surface for annealed and cold deformed conditions.

**Figure 13:**
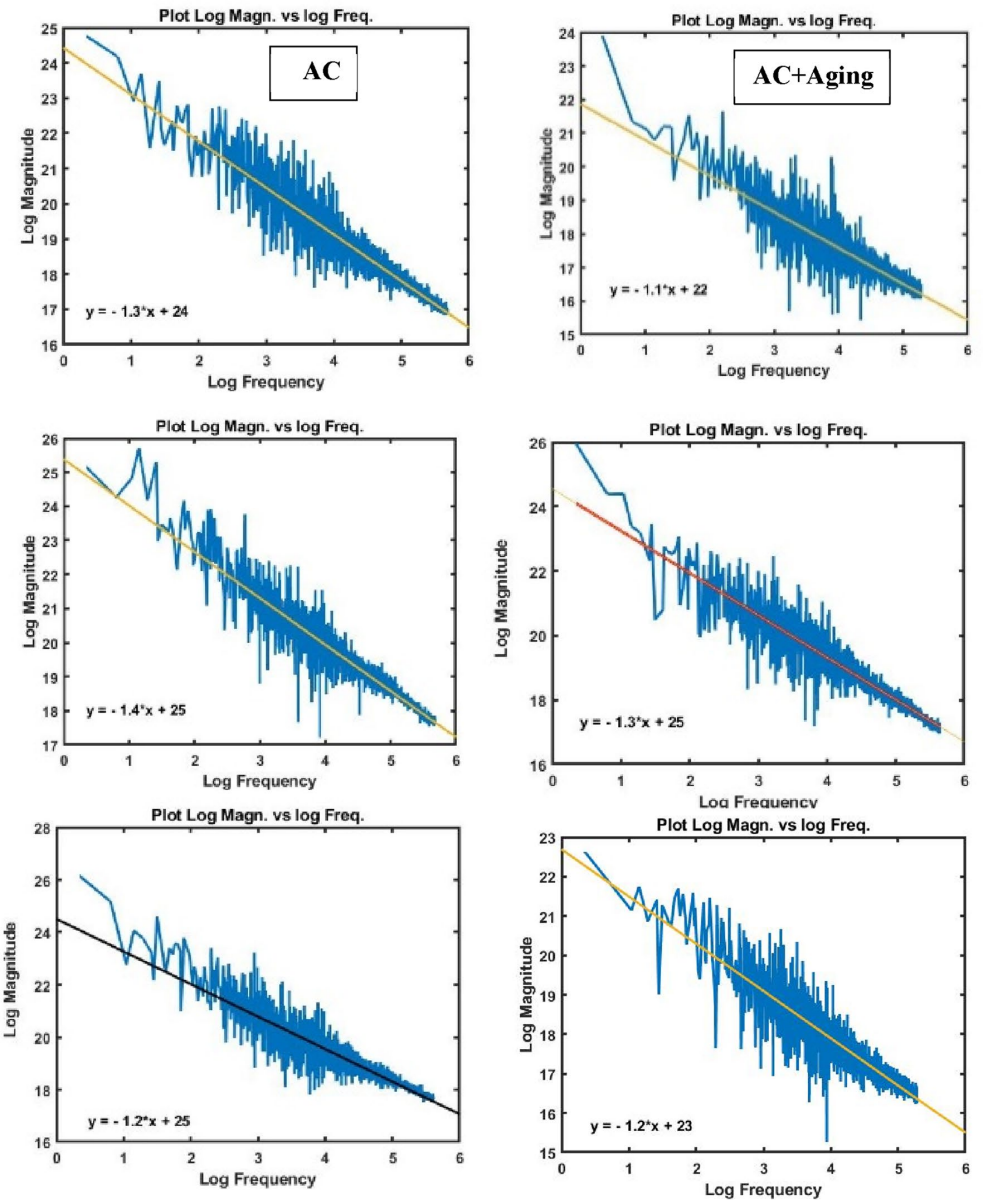
Different peaks of worn surface for AC and AC+Aging conditions.

Figures 12 and 13 demonstrate different peaks of worn surface for every conditions in qualitative manner. All figures emphasize existance of lamination (low peaks), plastic lines (ploughs) and hot peaks due to material defects.

**Figure 14:**
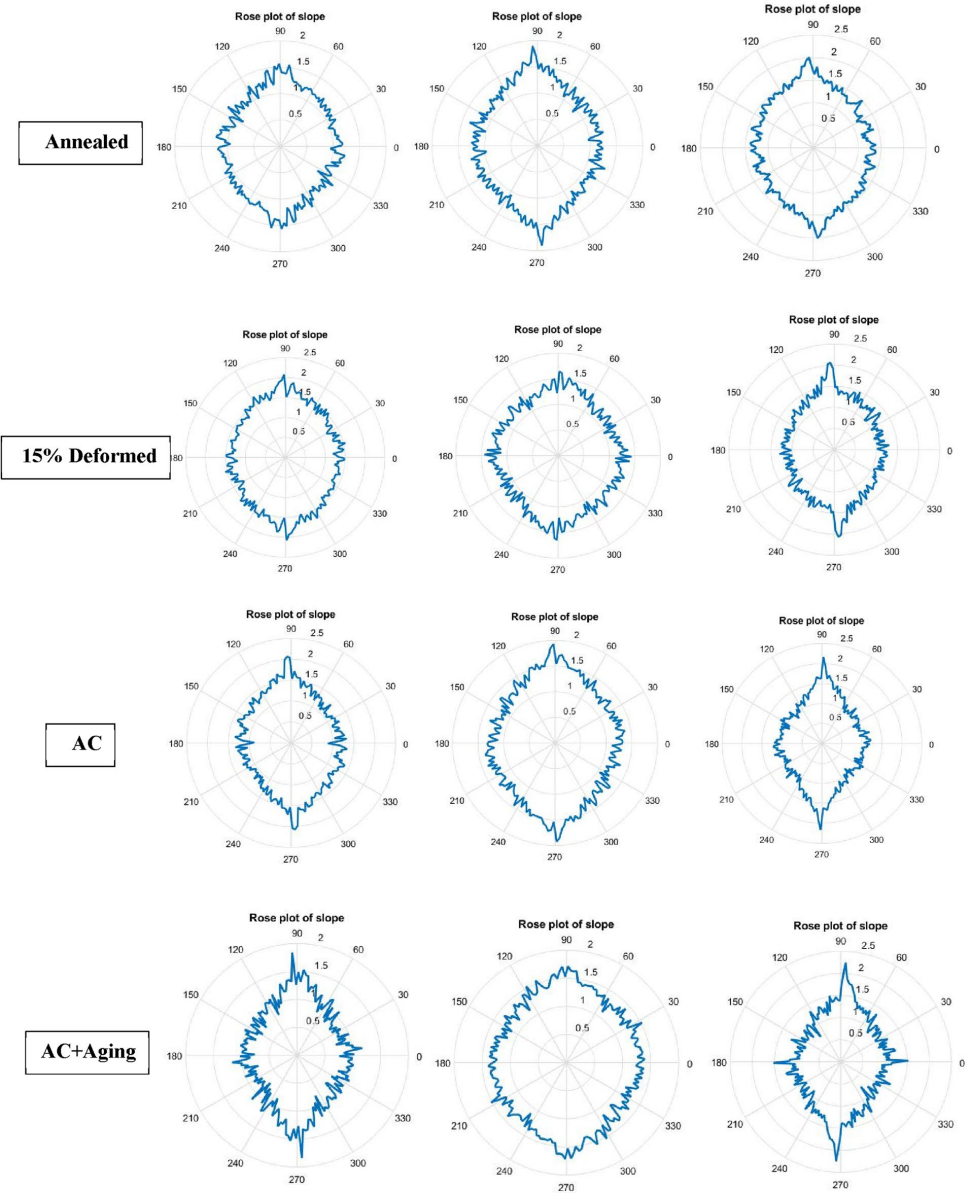
Rose plot of slope for all conditions.

**Figure 15:**
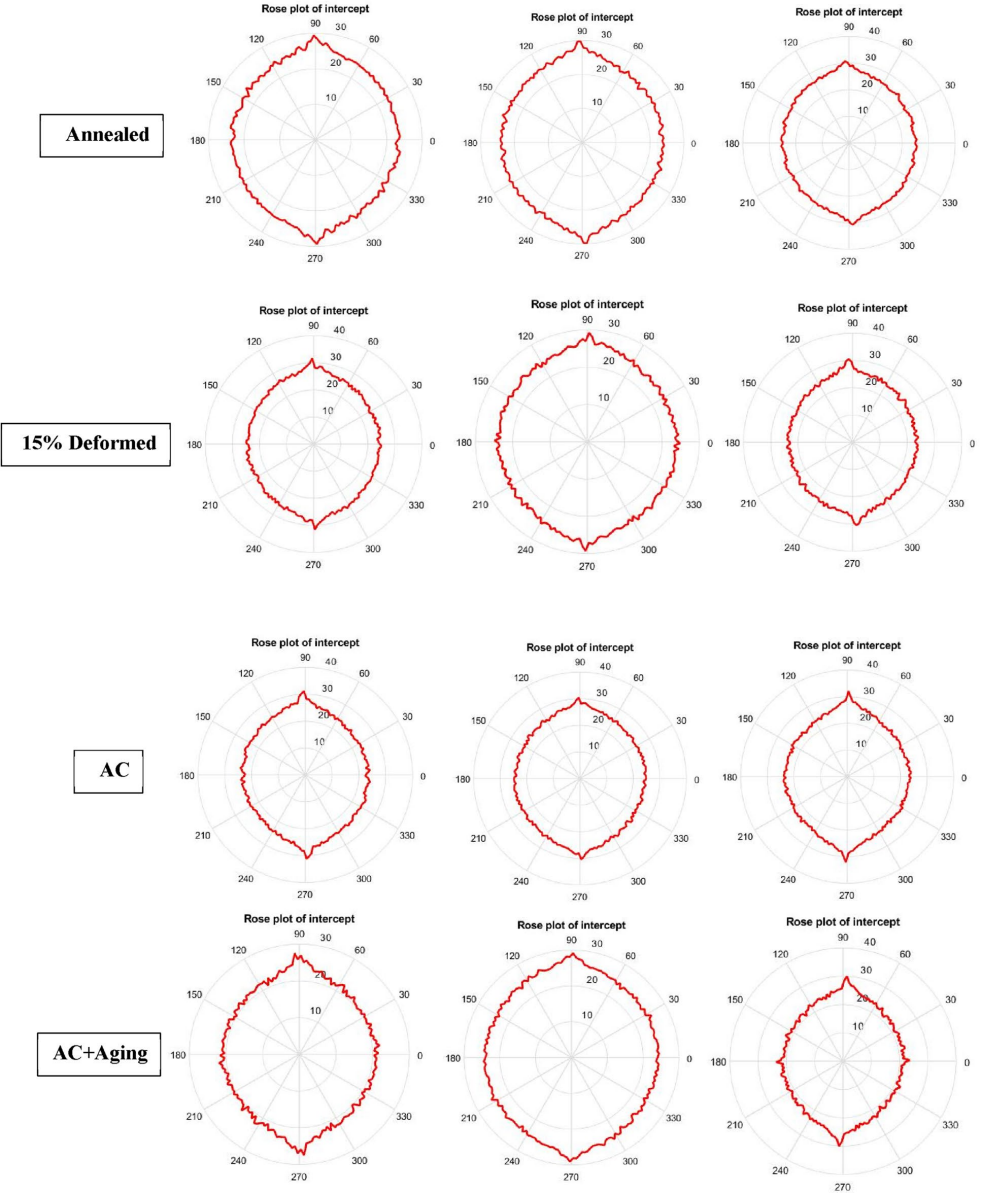
Rose plot of intercept for all conditions.

Figures 14 and 15 clarify different slopes and intercepts of TC21 Ti-alloy for the different conditions. It simplifies existance of material defects after every condiotin to determine arithmetic suface roughness.

**Figure 16:**
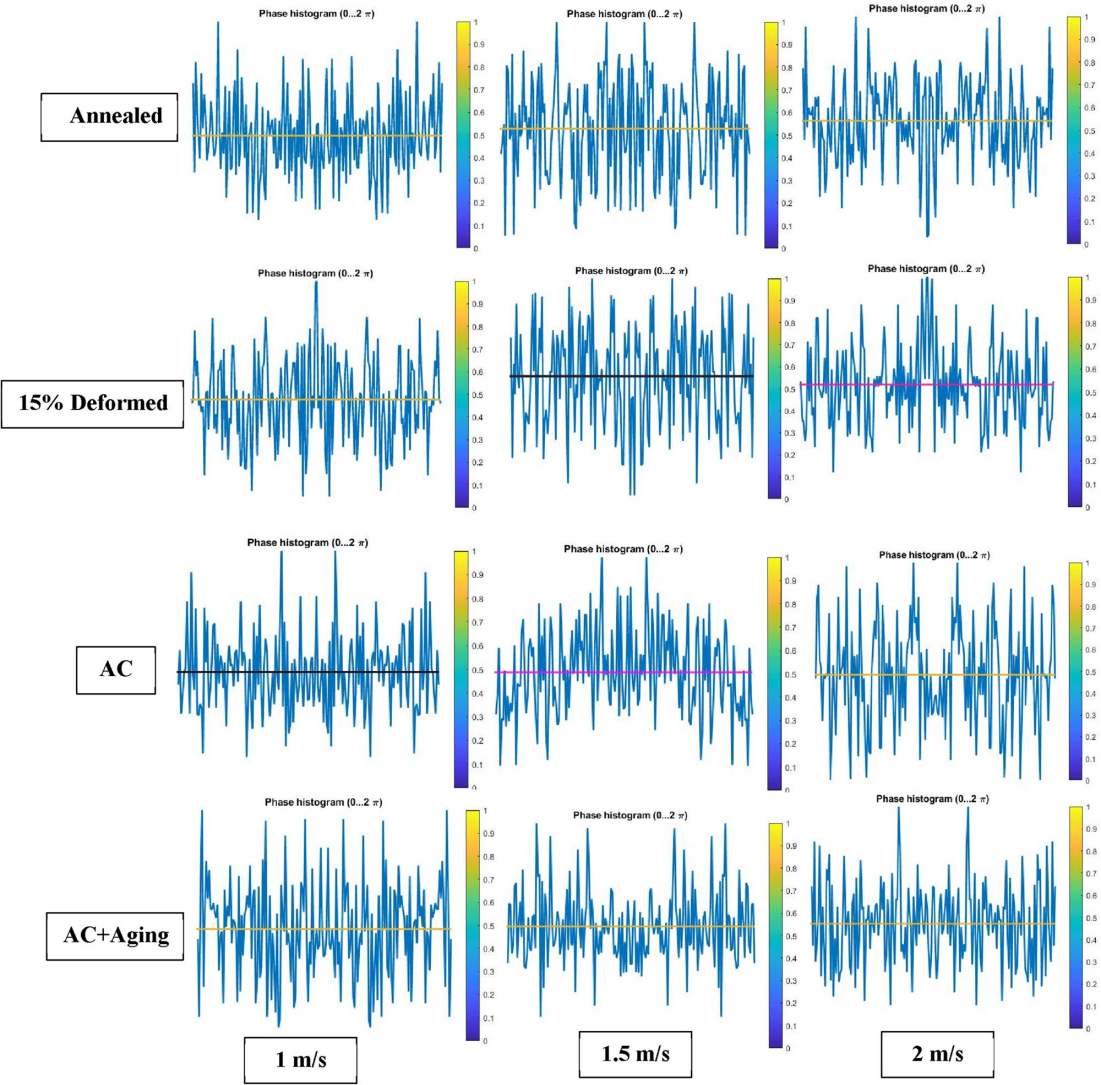
Surface roughness profile for the studied specimens at different conditions.

Figure 16 explains mean surface roughness deduced by MATLAB software to simulate the real surface roughness by Gwyddion. It is clear that different shapes of average surface roughness are in quit matching with different profiles as shown in Figs. 6, 7, 8 and 9. They consist of high peaks, low peaks and average peaks but in qualitative manner.

To understand behavior of worn surface and to determine the key parameter (hardness or velocity), it was necessary to construct mathematical modelling simulating wear rate versus velocity and material conditions (hardness) in quantitaive manner. It is absolutely crucial to investigate both parameters (hardness & velocity of wear test) on Abbott Firestone zones and to build a mathematical model that expresses Abbott Firestone zones in relation to hardness and velocity. CCD was used to illustrate hardness and velocity-related Abbott Firestone zones. Tables 3 and 4 display various limits for the parameters of hardness and velocity together with the associated Abbott Firestone zones (high peaks representing response 1, exploitation representing response 2, and voids representing response 3).

**Table 3:** Experimental CCD showing high peaks, exploitation and voids zones.

Std	Run	Factor 1	Factor 2	Response 1	Response 2	Response 3
		A: hardness, HV	B: velocity, m/s	High peaks zone, %	Exploitation zone, %	Voids zone, %
1	12	353 (Annealed)	1	13	86	1
2	13	425 (AC+Aging)	1	15	83	2
3	3	353 (Annealed)	2	22	76	2
4	2	425(AC+Aging)	2	29	70	1
5	5	353 (Annealed)	1.5	21	78	1
6	8	425 (AC+Aging)	1.5	23	74	3
7	7	389 (Cold deformed)	1	18	82	0
8	4	389 (Cold deformed)	2	20	77	3
9	11	389 (Cold deformed)	1.5	20	78	2
10	6	389 (Cold deformed)	1.5	20	78	2
11	9	389 (Cold deformed)	1.5	20	78	2
12	1	389 (Cold deformed)	1.5	20	78	2
13	10	389 (Cold deformed)	1.5	20	78	2

**Table 4:** Model summary statistics of high peaks zone.

Source	Sequential p-value	Std. dev.	R^2^	Adjusted R^2^	Predicted R^2^	PRESS	Recommendation
Linear	0.0018	2.20	0.7190	0.6628	0.3955	104.52	Suggested
2FI	0.2788	2.17	0.7552	0.6735	0.1262	151.09	
Quadratic	0.4289	2.18	0.8078	0.6704	− 0.9364	334.85	
Cubic	0.0002	0.4642	0.9938	0.9850	0.2759	125.22	Aliased

### Statistical analysis of wear rate

This section studies the effects of hardness and velocity on the Abbott Firestone zones of TC21 Ti-alloy, which represent as worn surfaces. The investigation and construction of the Abbott Firestone models were carried out using RSM. After proceeding with several trials using Design-Expert software, linear and 2FI models were proposed based on the statistical evaluation of several models, as shown in Tables 4, 5 and 6. The modified linear model is the best one for high peaks and exploitation zones. However, 2FI model for voids zone, which results in an elevated adjusted correlation factor. The software discovered also that for the data ranges obtained, the cubic model was aliased. R-squared values of high peaks, exploitation and voids zones are 0.7190, 0.8373, and 0.3853, respectively. However, adjusted R-squared values are 0.6628, 0.8048 and 0.1804, respectively.

**Table 5:** Model summary statistics of exploitation zone.

Source	Sequential p-value	Std. dev.	R^2^	Adjusted R^2^	Predicted R^2^	PRESS	Recommendation
Linear	0.0001	1.76	0.8373	0.8048	0.6661	63.33	Suggested
2FI	0.4220	1.78	0.8492	0.7989	0.5128	92.42	
Quadratic	0.1104	1.48	0.9196	0.8622	0.2009	151.58	
Cubic	0.0013	0.4642	0.9943	0.9864	0.3399	125.22	Aliased

**Table 6:** Model summary statistics of voids zone.

Source	Sequential p-value	Std. dev.	R^2^	Adjusted R^2^	Predicted R^2^	PRESS	Recommendation
Linear	0.2223	0.7678	0.2597	0.1117	− 0.6561	13.19	
2FI	0.2081	0.7375	0.3853	0.1804	− 1.5203	20.07	Suggested
Quadratic	0.5557	0.7689	0.4803	0.1091	− 4.2906	42.13	
Cubic	<0.0001	0.0186	0.9998	0.9995	0.9748	0.2003	Aliased

### ANOVA of Abbott Firestone zones

The statistical design tool known as ANOVA allows for differentiation of the individual impacts of the controlled variables. Finding statistically significant control factors is typically done using experimental data. Using DOE software and a response surface technique, the impacts of hardness (H) and velocity (V) on high peaks, exploitation, and voids zones were statistically studied. Empirical Abbott Firestone zones models were then developed based on these effects. The sequential F-test was used to evaluate the significance of the regression model. ANOVA-generated models of Abbott Firestone zones are displayed in Tables 7, 8 and 9. The model's F-values of 53.71, 162.40, and 3298.27 for high peaks, exploitation, and voids zones, respectively, provide evidence of its significance. A significant F-value is extremely unlikely to be caused by noise; which probability is only 0.01%. The predicted R^2^ value of 0.5682 for high peaks is nowhere near the adjusted R^2^ value of 0.9634, in which the difference is greater than 0.2, as one might typically anticipate. However, exploitation and voids zones are 0.8537, and 0.9748, respectively, which are as near to the adjusted R^2^ values as possible of 0.9878, and 0.9995; where the difference is smaller than 0.2. This can be a sign of a significant block effect or a potential issue with our model and/or data. Adeq precision values for high peaks, exploitation, and voids zones, respectively, of 28.4762, 49.0535, and 199.0782. It is best to indicate that the model may explore the design space by using a ratio greater than 4. The models' “P > F” values are less than 0.05, indicating that they are significant (high peaks, exploitation, and voids zones). This is advantageous since it shows how much the model's parameters affect the response (high peaks, exploitation, and voids zones). A, B, AB, A^2^, B^2^, and A^2^B are important terms in the model, among others. If the value is more than 0.1, the model terms are not significant. By eliminating the less significant model terms, the model might be improved. Final empirical Eqs. (3), (4) and (5) can identify high peaks, exploitation, and voids zones among the parameter range evaluated in terms of actual factors, hardness (H), velocity (V), and their multiplication products.

**Table 7:** ANOVA results of linear model (high peaks zone is response).

Source	Sum of squares	df	Mean square	F-value	P-value	
Model	169.76	6	28.29	53.71	< 0.0001	Significant
A-hardness	20.17	1	20.17	38.28	0.0008	
B-velocity	2.00	1	2.00	3.80	0.0993	
AB	6.25	1	6.25	11.86	0.0137	
A^2^	5.39	1	5.39	10.22	0.0187	
B^2^	7.10	1	7.10	13.48	0.0104	
A^2^B	30.08	1	30.08	57.10	0.0003	
Residual	3.16	6	0.5268			
Lack of fit	3.16	2	1.58			
Pure error	0.0000	4	0.0000			
Cor total	172.92	12				
Std. dev.	0.7258		R^2^		0.9817	
Mean	20.08		Adjusted R^2^		0.9634	
C.V. %	3.62		Predicted R^2^		0.5682	
PRESS	104.52		Adeq precision		28.4762

**Table 8:** ANOVA results of linear model (exploitation zone is response).

Source	Sum of squares	df	Mean square	F-value	P-value	
Model	188.53	6	31.42	162.40	< 0.0001	Significant
A-hardness	28.17	1	28.17	145.57	< 0.0001	
B-velocity	12.50	1	12.50	64.60	0.0002	
AB	2.25	1	2.25	11.63	0.0143	
A^2^	5.39	1	5.39	27.84	0.0019	
B^2^	12.22	1	12.22	63.16	0.0002	
A^2^B	14.08	1	14.08	72.79	0.0001	
Residual	1.16	6	0.1935			
Lack of fit	1.16	2	0.5805			
Pure error	0.0000	4	0.0000			
Cor total	189.69	12				
Std. dev.	0.4399		R^2^		0.9939	
Mean	78.15		Adjusted R^2^		0.9878	
C.V. %	0.5628		Predicted R^2^		0.8537	
PRESS	63.33		Adeq precision		49.0535

**Table 9:** ANOVA results of 2FI model (voids zone is response).

Source	Sum of squares	df	Mean square	F-value	P-value	
Model	7.96	7	1.14	3298.27	< 0.0001	Significant
A-hardness	2.00	1	2.00	5800.00	< 0.0001	
B-velocity	4.21	1	4.21	12194.50	< 0.0001	
AB	1.0000	1	1.0000	2900.00	< 0.0001	
A^2^	0.0016	1	0.0016	4.67	0.0832	
B^2^	0.6209	1	0.6209	1800.60	< 0.0001	
A^2^B	2.80	1	2.80	8129.67	< 0.0001	
Residual	1.33	1	1.33	3866.67	< 0.0001	
Lack of fit	0.0017	5	0.0003			
Pure error	0.0017	1	0.0017			
Cor total	0.0000	4	0.0000			
Std. dev.	0.0186		R^2^		0.9998	
Mean	1.78		Adjusted R^2^		0.9995	
C.V. %	1.05		Predicted R^2^		0.9748	
PRESS	20.07		Adeq Precision		199.0782	

3 $$\mathrm{High peaks }= -1477.31699+7.66280\times \mathrm{ H }+1103.44778\times \mathrm{ V }-5.63349\times \mathrm{ H }\times \mathrm{ V }-0.009918\times {\mathrm{H}}^{2} -6.41379\times {\mathrm{V}}^{2} +0.007330\times {\mathrm{H}}^{2} \times \mathrm{ V},$$

4 $$\mathrm{Exploitation}= +1078.70704-5.01233\times \mathrm{ H }-772.97325\times \mathrm{ V }+3.86034\times \mathrm{ H }\times \mathrm{ V }+0.006446\times {\mathrm{H}}^{2} +8.41379\times {\mathrm{V}}^{2} -0.005015\times {\mathrm{H}}^{2} \times \mathrm{ V}, $$

5 $$\mathrm{Voids}= + 568.71355-2.77741\times \mathrm{ H }-448.87555\times \mathrm{ V }+2.04645\times \mathrm{ H }\times \mathrm{ V }+0.003338\times {\mathrm{H}}^{2} +41.32567\times {\mathrm{V}}^{2} -0.002238\times {\mathrm{H}}^{2} \times \mathrm{ V}-0.111111\times \mathrm{ H }\times {\mathrm{V}}^{2},$$

### Graphical results of Abbott Firestone zones

**Figure 17:**
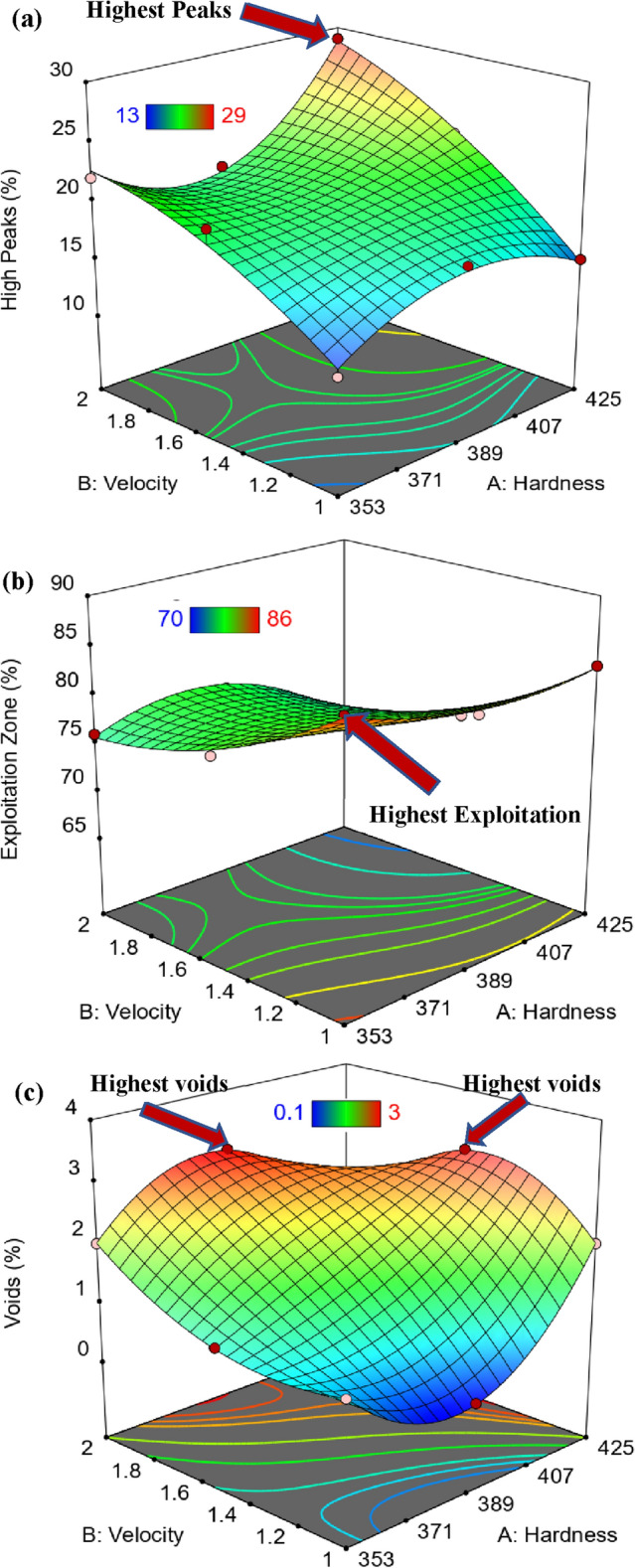
3D Surface plot of Abbott Firestone zone**s** (**a**) high peaks, (**b**) exploitation and (**c**) voids.

**Figure 18:**
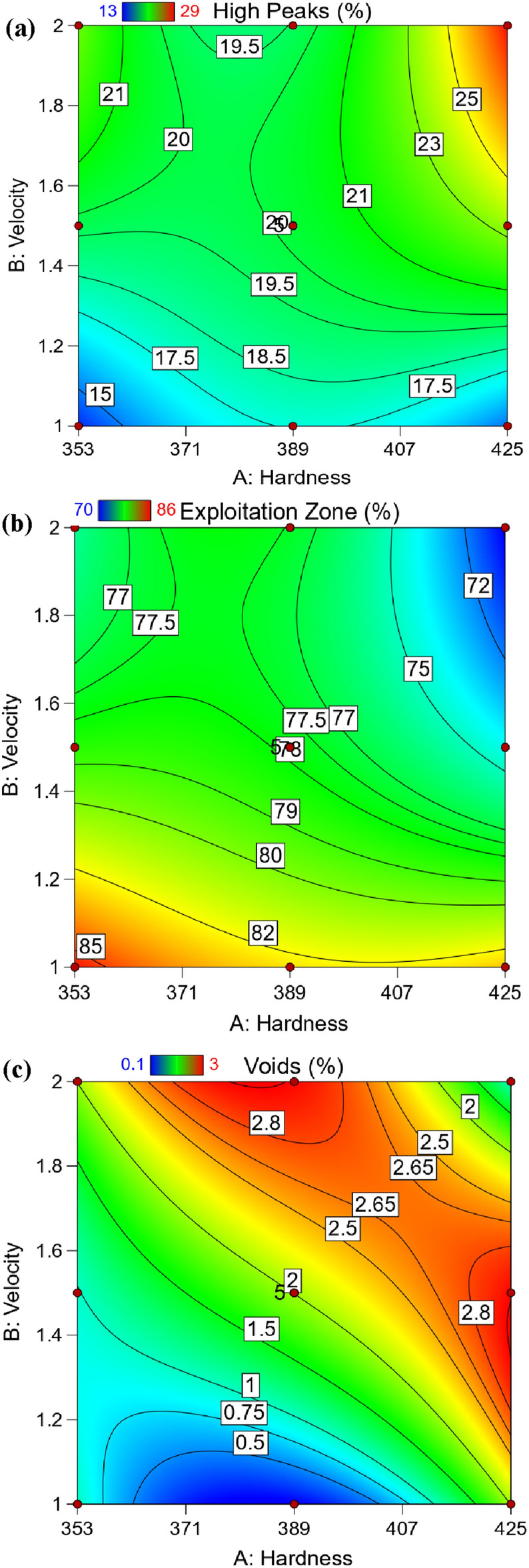
Contour plot of Abbott Firestone zones (**a**) high peaks, (**b**) exploitation and (**c**) voids.

**Figure 19:**
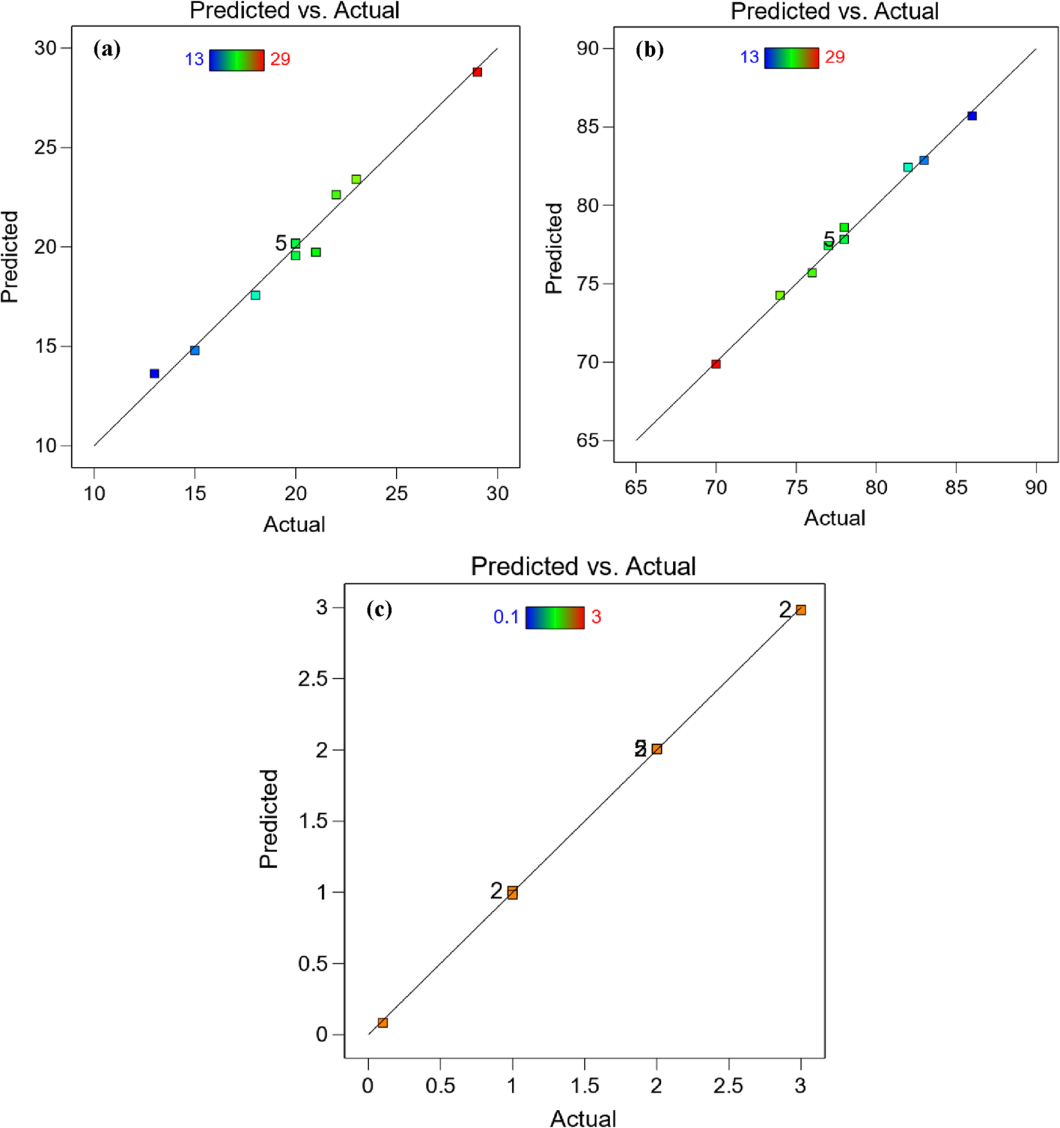
Relationship between actual and predicted Abbott Firestone zones (**a**) high peaks, (**b**) exploitation and (**c**) voids.

Building 3D surface and contour map using empirical equations is necessary to accurately follow the behavior of Abbott Firestone zones. Figure 17 displays the 3D surface plot of Abbott Firestone zones (high peaks, exploitation, and voids). The additional advantage of 3D visuals is possible to observe how the impact of one parameter changes when the value of another parameter changing. For instance, considering the effect of hardness (H) and velocity (V), it is clear that the velocity effect was stronger in high peak (Fig. 17a) and exploitation zones (Fig. 17b). Howevere, both hardness and velocity effect were stronger in voids zone (Fig. 17c). To predict the different values of Abbott Firestone zones it is a very useful to construct contour map as seen in Fig. 18. At increasing hardness and velocity, high peaks gradually increases (Fig. 18a), while at decreasing hardness and velocity exhibits an increase in exploitation zone (Fig. 18b). For medium hardness, increasing velocity gradually increases the voids zone (Fig. 18c). At low velocity, increasing hardness slightly increases high peaks making positive tipping point at middle value of hardness. At high velocity and low hardness, double increase of high peaks while increasing hardness at high velocity leads to slight decrease of high peaks (negative tipping). It is worthily mentioned that increasing both velocity and hardness, dramatically increases high peaks. Furthermore, exploitation zone is almost constant. Depth of voids are very low at both low harness and velocity. While it gives tipping at middle of hardness at low velocity and vice versa. By increasing velocity at high hardness, it gives high positive tipping and vice versa, however, it suddenly shows dramatic decrease by increasing both hardness and velocity. Figure 19 shows the relationship between actual and predicted Abbott Firestone zones, high peaks (Fig. 19a), exploitation (Fig. 19b) and voids (Fig. 19c).

## Conclusions

This study investigated worn surface topography and mathematical modeling of Ti-6Al-3Mo-2Sn-2Zr-2Nb-1.5Cr alloy using RSM. The following conclusions can be drawn from the results of the experiments and modeling:

1. Maximum hardness of 425 HV_20_ was obtained for AC+Aging specimen, while minimum hardness of 353 HV_20_ was reported for annealed specimen.
2. Due to the high hardness of AC+Aging specimen, it showed the lowest wear rate, while the annealed one had the highest wear rate. The aging process after solution treatment enhances considerably wear resistance reaching 98% compared to annealed specimen.
3. Average surface roughness (Ra) increases with increasing sliding velocity for all conditions except for AC+Aging condition where average surface roughness decreases while sliding velocity increases.
4. Models of Abbott Firestone zones (high peaks, exploitation, and voids) accurately predict wear behaviour of worn surfaces.
5. At low sliding velocity and hardness, the material gives the highest exploitation zone (86%). While at high velocity and hardness, the material gives the lowest exploitation zone (70%).
6. The anticipated results closely match the experimental findings, indicating that the developed models are successfully applied for predicting Abbott Firestone zones.
7. RSM model was used to find the best hardness and velocity of wear test for achieving the highest exploitation zones.

## Data Availability

All data generated or analyzed during this study are included in this published article.

## References

[1] Elshaer RN, Ibrahim KM (2020). Effect of cold deformation and heat treatment on microstructure and mechanical properties of TC21 Ti alloy. Trans. Nonferr. Met. Soc. China.

[2] Ahmed FS, El-Zomor MA, Ghazala MSA, Elshaer RN (2022). Effect of oxide layers formed by thermal oxidation on mechanical properties and NaCl-induced hot corrosion behavior of TC21 Ti-alloy. Sci. Rep..

[3] Elshaer RN, Abdelhameed M, Ibrahim KM, El-Shennawy M, Sobh A (2022). Static and fatigue characteristics of heat-treated Ti–6Al–3Mo–2Zr–2Sn–2Nb–1.5 Cr–0.1 Si alloy. Metallogr. Microstruct. Anal..

[4] Lin YC, Tang Y, Zhang X-Y, Chen C, Yang H, Zhou K-C (2019). Effects of solution temperature and cooling rate on microstructure and micro-hardness of a hot compressed Ti-6Al-4V alloy. Vacuum.

[5] Elshaer RN, Ibrahim KM (2022). Study of microstructure, mechanical properties, and corrosion behavior of as-cast Ni-Ti and Ti-6Al-4V alloys. J. Mater. Eng. Perform..

[6] Elshaer RN (2022). Effect of initial α-phase morphology on microstructure, mechanical properties, and work-hardening instability during heat treatment of TC21 Ti-alloy. Metallogr. Microstruct. Anal..

[7] Ahmed FS, El MA, Magdy Z, Ghazala SA, Elshaer RN (2022). Influence of α - phase morphology on mechanical characteristics, cycle oxidation, and hot corrosion behavior. Metallogr. Microstruct. Anal..

[8] Ibrahim KM, El-Hakeem AMM, Elshaer RN (2013). Microstructure and mechanical properties of cast and heat treated Ti–6.55 Al–3.41 Mo–1.77 Zr alloy. Trans. Nonferr. Met. Soc. China.

[9] Novák M, Náprstková N, Józwik J (2015). Analysis of the surface profile and its material share during the grinding inconel 718 alloy. Adv. Sci. Technol. Res. J..

[10] Rîpă M, Tomescu L, Hapenciuc M, Crudu I. Tribological characterisation of surface topography using Abbott-Firestone curve. *Ann. Univ. Dunǎrea Jos Galati, Fascicle VIII, Tribol.*, 208–212 (2003).

[11] Tomescu L, Ripa M, Georgescu C (2001). Analysing Abbott curve for composites with polymeric matrix and fibbers. Tribol. Ind..

[12] Torrance AA (1997). A simple datum for measurement of the Abbott curve of a profile and its first derivative. Tribol. Int..

[13] Sosa M, Björklund S, Sellgren U, Olofsson U (2015). In situ surface characterization of running-in of involute gears. Wear.

[14] Sosa M, Sellgren U, Björklund S, Olofsson U (2016). In situ running-in analysis of ground gears. Wear.

[15] Affatato S, Ruggiero A, De Mattia JS, Taddei P (2016). Does metal transfer affect the tribological behaviour of femoral heads? Roughness and phase transformation analyses on retrieved zirconia and Biolox® Delta composites. Compos. B Eng..

[16] Mathia TG, Pawlus P, Wieczorowski M (2011). Recent trends in surface metrology. Wear.

[17] Bruzzone AAG, Costa HL, Lonardo PM, Lucca DA (2008). Advances in engineered surfaces for functional performance. CIRP Ann..

[18] Kara F, Küçük Y, Özbek O (2023). Effect of cryogenic treatment on wear behavior of Sleipner cold work tool steel. Tribol. Int..

[19] Elshaer RN, El-Fawakhry MK, Farahat AIZ (2021). Behavior of carbon steel machine elements in acidic environment. Metallogr. Microstruct. Anal..

[20] Elshaer RN, El-Fawakhry MK, Mattar T, Farahat AIZ (2022). Mathematical modeling of wear behavior and Abbott Firestone zones of 0.16 C steel using response surface methodology. Sci. Rep..

[21] Saravanan I, Perumal AE, Vettivel SC, Selvakumar N, Baradeswaran A (2015). Optimizing wear behavior of TiN coated SS 316L against Ti alloy using response surface methodology. Mater. Des..

[22] Nas E, Özbek O, Bayraktar F, Kara F (2021). Experimental and statistical investigation of machinability of AISI D2 steel using electroerosion machining method in different machining parameters. Adv. Mater. Sci. Eng..

[23] Manoj IV, Soni H, Narendranath S, Mashinini PM, Kara F (2022). Examination of machining parameters and prediction of cutting velocity and surface roughness using RSM and ANN using WEDM of Altemp HX. Adv. Mater. Sci. Eng..

[24] Mohammed Razzaq A, Majid DL, Ishak MR, Muwafaq Basheer U (2020). Mathematical modeling and analysis of tribological properties of AA6063 aluminum alloy reinforced with fly ash by using response surface methodology. Crystals.

[25] Abdelmoneim A, Elshaer RN, El-Shennawy M, Sobh AS (2023). Modeling of wear resistance for TC21 Ti-alloy using response surface methodology. Sci. Rep..

[26] Chauhan SR, Dass K (2013). Dry sliding wear behaviour of titanium (Grade 5) alloy by using response surface methodology. Adv. Tribol..

[27] Meddah S, Bourebia M, Oulabbas A, et al. Prediction of the Friction Coefficient of 13Cr5Ni2Mo Steel Using Experiments Plans-Study of Wear Behavior. In *Proceedings of the International Conference on Industrial Engineering and Operations Management*, 3, 1129–1135 (2019).

[28] Elshaer RN, El-Deeb MSS, Mohamed SS, Ibrahim KM (2022). Effect of strain hardening and aging processes on microstructure evolution, tensile and fatigue properties of cast Ti-6Al-2Sn-2Zr–2Mo-1.5 Cr-2Nb-0.1 Si Alloy. Int. J. Met..

